# Role of metal nanomaterials in wound healing - a review

**DOI:** 10.3389/fbioe.2025.1665218

**Published:** 2026-03-12

**Authors:** Said El Turk, Dileep Chekkaramkodi, Aswathi Ram P, Amr Soliman, Andreas Schiffer, Lianxi Zheng, Haider Butt

**Affiliations:** 1 Department of Mechanical and Nuclear Engineering, Khalifa University of Science and Technology, Abu Dhabi, United Arab Emirates; 2 Seagate Technology, Derry, United Kingdom; 3 Advanced Digital and Additive Manufacturing (ADAM) Center, Khalifa University of Science and Technology, Abu Dhabi, United Arab Emirates

**Keywords:** wound healing, metallic nanomaterials, metal ions, tissue regeneration, wound patches

## Abstract

Wounds pose a significant burden in a patient’s life. Nanotechnology has developed a new era of wound healing through the introduction of nanoparticles. This paper reviews the performance of various metallic nanoparticles, like silver, gold, titanium, magnesium, cerium, and zinc in bacterial reduction, inflammation control, and wound healing when incorporated into wound healing patches. MNPs exhibit antibacterial and anti-inflammatory properties, primarily through reactive oxygen species generation and the release of metallic ions, leading to bacterial cell wall disruption and nutrient deprivation. Their presence at the wound site accelerates healing, enhances wound closure, and promotes cell proliferation. For instance, gold nanoparticles with hydrogels have shown an effectiveness of more than 95% against certain strains and an enhancement in wound healing and closure. Additionally, copper nanoparticles have shown an effectiveness of more than 99% against certain strains and an advancement in the healing process. The review elaborates on the diverse hydrogels, antibacterial, and wound-healing mechanisms of different nanoparticles, as well as future pathways. However, concerns regarding the long-term toxicity of MNPs and immune responses due to prolonged exposure to metal ions remain, which is extensively discussed in the review. Additionally, research on MNPs beyond gold and silver nanoparticles is limited, necessitating further studies to understand their mechanisms and efficacy in wound healing applications.

## 1 Introduction

A wound results from trauma, bodily injury, or impacts that lead to the rupture of the skin’s outermost layer. Wound patches are protective coverings applied to injuries to prevent further external harm (Teodoro et al., 2022). These patches not only play a crucial role in protecting wounds, but also alleviate pain and safeguard wounds from bacterial infections (Sharma et al., 2024; White and Valdehita, 2009). Moreover, wound patches sustain a moist environment near the wounds, facilitating efficient and rapid healing and expediting their closure (Elangwe et al., 2022).

**FIGURE 1:**
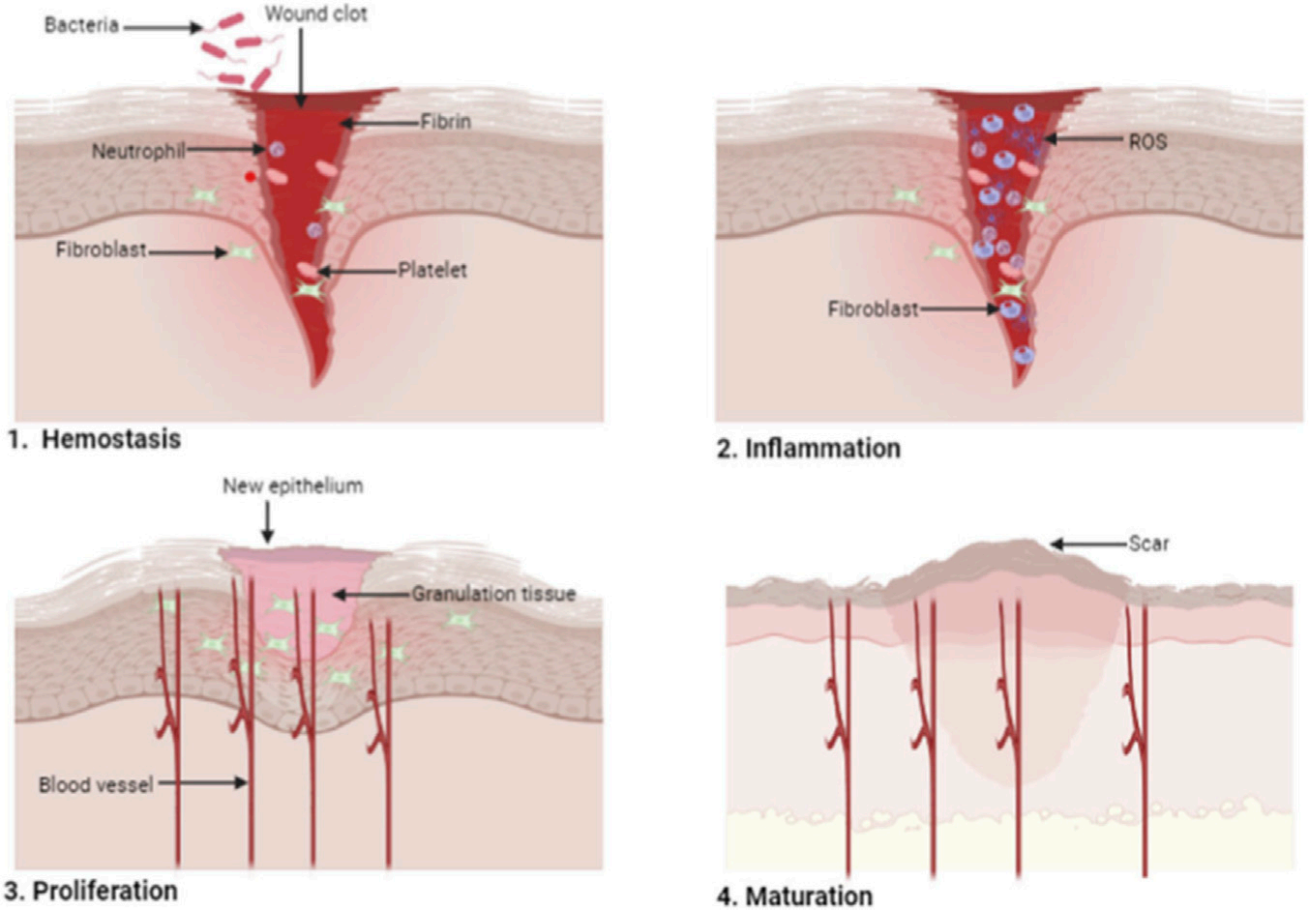
Schematic illustration showing different stages of wound healing (Alven and Aderibigbe, 2024). Reproduced from Alven, S.; Aderibigbe, B. A. Chitosan-Based Scaffolds Incorporated with Silver Nanoparticles for the Treatment of Infected Wounds, Pharmaceutics 2024, 16, 327. https://doi.org/10.3390/pharmaceutics16030327. Licensed under CC BY 4.0. (https://creativecommons.org/licenses/by/4.0/).

The wound healing process is a physiological process that consists of several sequential cellular and molecular events that promote skin regeneration (Sheikh-Oleslami et al., 2023). The healing process is divided into four key stages: the hemostasis phase, the inflammatory phase, the proliferative phase, and the remodeling phase (maturation), as shown in Figure 1 (Rajendran et al., 2018). During the hemostasis phase, which takes from seconds to hours, clot formation and vasoconstriction occur. The inflammation phase, which can take hours to days, is the phase in which immune cells are recruited, and microbes are eliminated. The proliferation phase, which can take days to a week, involves the formation of new tissues and the growth of the blood vessels. The remodeling phase, which can take from a week to months, involves the deposition of collagen and wound closure. Hemostasis occurs within seconds to hours after injury and is characterized by clot formation and vasoconstriction. The subsequent inflammatory phase, lasting several hours to days, is distinguished by the recruitment of immune effector cells and the eradication of microbes. The proliferation phase, which spans several days to approximately 1 week, involves the formation of new tissues and the growth of blood vessels. The final remodeling phase extends from 1 week to several months and entails collagen deposition, extracellular-matrix remodeling, and progressive wound contraction leading to definitive closure. Typically, wounds in healthy individuals tend to heal within 2–3 weeks. However, certain factors, such as poor blood supply and the existence of foreign bodies at the wound site, which affect tissue repair, might extend the timeline of the healing process (Rajendran et al., 2018).

The fundamental operational concepts of wound patches are predicated on three primary considerations: (i) preserving a wound-moist environment facilitates tissue regeneration (Elangwe et al., 2022); (ii) the cessation of rapid bleeding commences the healing phase (Yang et al., 2021); (iii) the biocompatibility of the patches material facilitates a natural healing process with minimal adverse responses (Elangwe et al., 2022; Gumenyuk et al., 2023).

Bacteria can be eliminated when they experience induced oxidative stresses and disruption in their walls (Qin et al., 2024; Zheng Y. et al., 2024; Ge et al., 2024). Antibiotics, such as Penicillin, work on inhibiting the enzymes that synthesize cell wall components, which compromise the integrity of the bacterial cell walls and make them vulnerable to rupture (Kohanski et al., 2010). Bacteria can also be eliminated by reactive oxygen species (ROS), which apply oxidative stresses on their DNA, lipids, and proteins, causing eventual death (Storz and Imlayt, 1999; Sies, 2017). Also, damaging the bacteria’s membrane or interfering with the synthesis of their DNA and proteins, which is what antimicrobial drugs do, can eventually lead to the death of bacteria (Maisonneuve and Gerdes, 2014).

The invasion of pathogenic bacteria into open wounds will impact the surrounding skin cells, leading to an abnormal wound environment that impairs healing (Ye et al., 2025). Infectious wounds are being healed with the introduction of highly biocompatible and ROS-responsive hydrogels, which also promote tissue regeneration (Huang et al., 2022; Uberoi et al., 2024).

The formation of Thrombus on blood-contacting biomaterials can pose risks to the patient’s life and affect the performance of the device. Hence, intensive research has been conducted on the development of superhydrophobic or super-lubricated coatings for specific surface functionalization (Wang et al., 2023). Along with that, hemostatic biomaterials with porous structures and strongly adhesive hydrogels can be In addition, porous hemostatic biomaterials and highly adhesive hydrogels can be employed to achieve rapid responsive hemostasis (Singh et al., 2020; Shin et al., 2021).

Despite the fact that progress has been made in cancer therapies, the therapeutic options for managing bone metastases remain limited. New nanomaterial-based therapies possess multifunctional capabilities that are efficient for multimodal bone metastasis therapy, offering a benefit of targeted designs that provide outstanding penetration and immune regulation in the tumor location (Hao et al., 2024).

Enamel, a biological tissue in humans, has a complex architecture. Carboxymethyl chitosan and lysozyme (CMC/LYZ) nanogels loaded with amorphous calcium phosphate (ACP) were produced and may be promptly applied on the tooth's surface as a treatment for enamel remineralization (Zhou et al., 2025). These nanogels could also be integrated with agents that possess antibacterial properties, diminishing the Bacterial viability in the oral cavity (Zhou et al., 2025). Thus, it was proposed that incorporating antibacterial agents into biomimetic remineralization systems (ACP@CMC/LYZ nanogels) would generate new bifunctional nanogels.

The traditional methodology of using antibiotics is now facing high challenges due to the increasing cases of antibiotic resistance. Hence, metallic nanoparticles (MNPs) with antimicrobial properties have a high potential of being used as an alternative to antibiotics (Sheikh-Oleslami et al., 2023). Utilizing MNPs in wound patches can expedite healing and decrease the risk of recurrent infections. For instance, gold, copper, zinc oxide, and silver nanoparticles exhibit features such as enhanced healing, antibacterial efficacy, and drug transport capabilities (Mendes et al., 2022). MNPs facilitate the appropriate advancement of each healing phase, enhancing both efficacy and speed of recovery. Moreover, MNPs infiltrate the wound and engage in biological interactions, resulting in effectively regulated healing (Palani et al., 2024).

The characteristics of MNPs in wound healing are contingent upon many mechanisms (Chinnaiyan et al., 2024; Aminzai et al., 2024). As an example, iron nanoparticles release metallic ions that possess antibacterial properties and counteract bacterial resistance, hence facilitating healing (Lu Z. et al., 2023). Also, they combat infections by suppressing protein synthesis, peroxidizing cell membranes, and destroying bacterial nucleic acids (Salvo and Sandoval, 2022). MNPs can facilitate treatment to wounds by exerting therapeutic effects, including modulation of growth factors and antimicrobial effects, at the injury site (Salvo and Sandoval, 2022).

While recent advancements in wound healing patches have brought significant improvements, they still face several limitations (Shariatzadeh et al., 2024; Wang C. et al., 2024). For instance, patches made of biopolymers have weak mechanical properties that negatively affect their durability and effectiveness (Ndlovu et al., 2021). Also, some wound patches degrade rapidly, which limits their effectiveness in healing long-term wounds (Alven and Aderibigbe, 2021). Furthermore, the traditionally used patches face challenges in adhering to the wound site, which causes pain and possibly skin damage when removed or replaced (Sheokand et al., 2023). Utilizing MNPs in wound healing not only promotes effective wound healing but also helps inhibit future secondary bacterial infections (Palani et al., 2024). MNPs can be integrated with other types of materials, such as hydrogels, enabling the creation of advanced wound dressings with enhanced properties (Wang Y. et al., 2024). Moreover, the incorporation of MNPs in hydrogel wound dressings holds significant potential for improving wound treatment precision and efficacy (Wang Y. et al., 2024).

## 2 Scientific mechanisms

### 2.1 Antibacterial activity *via* metal ion release

The release of metallic ions in MNPs is a crucial mechanism for fighting bacteria. The release of ions mechanism is associated with widely silver nanoparticles, as shown in Table 1. The release of ions is generally associated with the surface reactions and corrosion of MNPs (Kessler et al., 2022). MNPs release ions when they are in contact with oxygen or moisture. The release process is affected by several factors, including the existence of other types of ions, the surface coating of nanoparticles, the pH, the nanoparticle’s size, concentration, and the conditions of the environment (Domingos et al., 2015; Mulenos et al., 2020; Zhang et al., 2011). The release rate of ions can be controlled using an electrochemical methodology, where applying voltage and current influences the release rate (Tseng and Liao, 2011). This approach enables the fabrication of bioactive nanocomposites that are controlled by release rate and time (Chudobova et al., 2015).

**TABLE 1:** A comparative table of nanoparticles, on antibacterial mechanisms, toxicity, and biocompatibility.

Nanoparticle	Antibacterial mechanism	Toxicity	Biocompatibility	Ref
Silver NPs	Metal ions release/ROS Generation/Cell wall disruption	Dose-dependent cytotoxicity; liver and kidney accumulation; Oxidative stress >50 μg/mL	Used in wound dressings. Safe at lower concentrations	Kim et al. (2009), Marambio-Jones and Hoek (2010), Ahamed et al. (2010)
Gold NPs	Cell membrane disruption/Interaction with biomolecules within bacteria/Photothermal effect/ROS generation	Interaction with mammalian biomolecules; Causes Oxidative stress, Inflammation, and genotoxicity at high doses	Safest at lower concentrations (from 100 to 200 μL/mL); AuNPs–Hb corona formation increase appreciably at higher concentrations	Zhao et al. (2010), Ahmed et al. (2020), Mahmoud et al. (2018), Kumar et al. (2020)
Zinc Oxide NPs	Cell membrane disruption/Release of antimicrobial ions/Direct interaction with microorganisms/ROS generation	Dose-dependent toxicity; cytotoxicity starts at concentrations of 10 and 25 μg/mL; ZnO NP (0.1–100 μg/mL) showed to be genotoxic	FDA recognized non-harmful material; widely used in biomedical imaging, biosensing, gene delivery, and drug delivery	Ali et al. (2018), Zhang et al. (2013); Heim et al. (2015), Abebe et al. (2020)
Titanium Dioxide NPs	Depolarization of cell membranes/Photocatalysis/Metal ions release/enhancement of the effectiveness of antimicrobial compounds	DNA disruption; Interaction with internal organs causes gastric disorders, Liver swelling and cardiac injuries; Higher concentration leads to various chronic diseases	Medicinal applications; chemically stable	Jackson et al., 2016; Manzoor et al. (2024), Serov et al. (2024), Kubacka et al. (2014), Khater et al. (2020)
Copper Oxide NPs	Rupture of cell membranes/ROS release/Metal ions release/Photocatalytic activity/Dose dependent	Causes mitochondrial dysfunction; induction of apoptosis; lipid peroxidation; protein denaturation; death receptor activation. IC30∼ 25–50ppm	No deleterious effects on living cells. CuNPs concentration of 35 nmol/mg shows more biocompatibility	Sajjad et al. (2023), Patlolla et al. (2009), Gudkov et al. (2024)
Cerium Oxide NPs	Surface contact with bacteria/ROS generation/Hypobromous acid formation/Interference with transportation	No key takeaways can be drawn on their toxicity due to the different behavior of different CeO_2_ samples	Potential use in surgical implants; reports state on high dose usage, nanoparticles migrating from implant sites and deposit in liver, lungs, spleen and kidneys	Thill et al. (2006), Leung et al. (2015), Zheng et al. (2024c)
Magnesium Hydroxide NPs	Cell wall disruption/Metal ions release	Inflammatory response MgO nanoparticles at doses of 250 and 500 μg.mL^-1;^ Dose-dependent pulmonary toxicity and genotoxicity	Considered safe *in vivo* and *in vitro* at lower concentrations; safe at <250 μg/mL suitable for drug encapsulation, water purification and clinical uses.; Higher concentrations may lead to cancer	Mazaheri et al. (2019), Dong et al. (2014), Xia et al. (2024)

Small concentrations of MNPs utilize various mechanisms to inhibit bacterial strains and exhibit outstanding performance. It has been shown that the antibacterial activity of NPs is based on and proportional to the release of ions, although other mechanisms can be involved as well (Ivask et al., 2014). By the release of metallic ions, MNPs can damage the microbial enzymes, change the membrane’s integrity, and form ROS. Their antimicrobial properties are generally due to their ability to interact with cellular biomacromolecules and components such as DNA and RNA, penetrate the cell walls of bacteria, and build up in the periplasmic space (Chudobova et al., 2015; Rajkumari et al., 2023). The ability of MNPs to inhibit the growth and kill off bacteria by disrupting the cellular processes has been successfully proven (VASHISTHA et al., 2024).

When it comes to disrupting the bacterial cell wall, the metal ions released can interact with the cell walls and cause structural damage. That is because the released ions can bind to the components of the cell wall, which causes increased permeability and cell destruction (Rajkumari et al., 2023). The ions released from MNPs can also disrupt the function of bacterial proteins by binding to them. This binding interaction can inhibit the essential activities of enzymes or denature proteins, which will finally lead to cell death (Rajkumari et al., 2023). Also, the ions released can interact with the DNA of bacteria and damage it, which can inhibit replication. This interaction can cause the eventual death of bacteria (Rajkumari et al., 2023).

Wigginton et al. observed that more than half of bacterial proteins, both enzymatic and non-enzymatic, have a high affinity for NPs and metal ions (Wigginton et al., 2010). Because of ion transport in cells, the NPs-nucleic acids interaction is the primary antibacterial mechanism. Chatterjee et al. found that *S. aureus* and *E. coli* exhibited DNA condensation after being treated with Ag NPs. Furthermore, their data suggested that the DNA of *E. coli* was more sensitive than *S. aureus* (Chatterjee et al., 2015).

The medium in which metal NPs are disseminated has a substantial influence on the ions they release. Levard et al. discovered that chloride in culture medium or seawater can promote the release of ions from AgNPs (Levard et al., 2013). Despite initially increasing bactericidal activity, this release shortens the life of the NPs and, as a result, lowers their antimicrobial effectiveness over time.

### 2.2 Antibacterial activity through ROS generation

Another significant mechanism underlying metal NP-mediated antibacterial action is the generation of ROS. ROS can disturb bacterial cells, whether they are created outside or inside the cell (Wang et al., 2014). High ROS levels can harm the cellular membrane, damage nucleic acids and proteins and ultimately causing cell death. Metal nanomaterials enhance the generation of ROS in the cells of bacteria causing DNA destabilization through intercalation between pyrimidine and purine bases (Sirelkhatim et al., 2015). As a result, transduction and metabolic signals are changed, and cell proliferation is suppressed.

ROS amount by nanoparticles can depend on the size. Mujeeb et al. observed that Ag–Cu nanocomposites (Ag-CuNCs), fabricated by utilizing *Olax scandens* leaf extract, display better antimicrobial activity when compared to Ag NPs, associated with the increased generation of ROS (Mujeeb et al., 2020). Wang et al. reported that the bactericidal effect of Ag/CeO_2_ NPs is mainly due to intracellular ROS generation and cell wall and membrane rupture in *E. coli* (Wang et al., 2014) and not because of the Ag ion release.

The released metallic ions can generate ROS, such as hydroxyl radicals, by undergoing redox reactions (Kessler et al., 2022). For example, the reaction shown below is a common redox reaction where ferrous ions react with hydrogen peroxide to produce ROS and hydroxyl radicals (Shahnazarova et al., 2024).

$${\text{Fe}}^{2+}+H_{2}O_{2}\to {\text{Fe}}^{3+}+\text{OH}˙+{\text{OH}}^{-}$$

The amount of ROS produced can be fatal to bacterial cell survival by interfering with bacterial metabolism and signal pathways (Paiva and Bozza, 2014). ROS can strengthen the host immune system’s bactericidal ability by encouraging the formation of neutrophil extracellular traps, which aid in the removal of pathogens (Paiva and Bozza, 2014).

### 2.3 Antibacterial activity through physical contact

MNPs can have physical interaction with the intracellular components and cell membrane/walls. The destruction of cell walls of bacteria is fatal since their inner body is protected from the external environment. The NPs-bacterial interaction causes membrane damage because of their cells adsorption and penetration (Thill et al., 2006). Adsorption of NPs promotes cell wall depolarization, which modifies the wall's negative charge and makes it more permeable. As a result, the cell wall is damaged, and ROS are generated (Ramalingam et al., 2016). Studies show that the AgNPs will compromise the cell wall structure and enhance the ion permeability to the cytosol (Sukhanova et al., 2018).

Researchers have shown that the Mg(OH)_2_NPs can cause cell death through electrostatic adsorption without entering the cell wall (Pan et al., 2013). Smaller nanoparticles can cause antibacterial activity through membranes and their penetration through the cell walls (Mukha et al., 2013; Franco et al., 2022).

### 2.4 Antioxidant and anti-inflammatory effects

MNPs (such as gold and silver) natively exhibit antioxidant capabilities and do not necessitate any functional alteration to possess these qualities. Nanomaterials exhibit oxidant activity through their capacity to generate ROS, while their ability to scavenge ROS is characterized as antioxidant activity (Patel et al., 2024). ROS includes radicals that are free of oxygen, along with additional molecules containing a minimum of one oxygen atom with one or more unpaired electrons that can exist individually. The existence of electrons that are unpaired in these entities renders them very unstable and reactive (Patel et al., 2024).

ROS, including hydroxyl and hydrogen peroxide radicals, have a high degree of reactivity and may cause the oxidation of vital biological components of the bacteria, such as nucleic acids and proteins, which leads to the rupture of bacterial cell walls and eventual death of the bacteria. This serves as a basis for utilizing ROS-generating compounds as antibacterial agents (Liu et al., 2023).

MNPs can generate ROS using several mechanisms, such as interaction with light and photocatalytic activity. When MNPs interact with light, their local surface plasmon resonance excites. This excitation promotes the transfer of electrons from one molecule to another, leading to the generation of ROS (Xu et al., 2023; da Silva et al., 2020). Nanoparticles exhibiting photocatalytic activity possess the capability to induce non-specific cell death through the generation of ROS upon interaction with biological entities (Sahoo et al., 2023). The generation process is initiated when the MNPs come in contact with light, which excites the electrons in the MNPs. The excitation causes the generation of electron-hole pairs, and upon their interaction with water and oxygen molecules in redox reactions, ROS are generated (Malec et al., 2023).

The antioxidant system of a living organism is defined as an oxidative defense mechanism. This defense mechanism comprises chemicals present in lower concentrations than oxidizable substrates and can inhibit or avert oxidation within the body (Liu et al., 2023). The antioxidant defense system should not substantially reduce ROS levels, but rather allow for adequate ROS levels to be sustained for their appropriate tasks (Liu et al., 2023).

Considering the strong correlation between ROS and inflammation, biomaterials designed for ROS scavenging have been developed to mitigate inflammation and safeguard the host from damage resulting from dysregulated inflammatory responses. ROS-scavenging biomaterials reduce inflammation by directly or indirectly regulating the synthesis and removal of reactive oxygen species (ROS). Conventional anti-inflammatory medications, including corticosteroids and non-steroidal anti-inflammatory medicines, may induce adverse effects on several organs, such as gastrointestinal and cardiovascular issues, as well as renal failure (Liu et al., 2023). ROS-scavenging biomaterials are robust adjuncts to existing therapeutic pharmaceuticals in the management of inflammation (Liu et al., 2023).

Excessive production of ROS causes inflammation, which can lead to tissue injury and excessive oxidative stress. Nanomaterials can scavenge ROS to reduce these inflammatory responses and oxidative stress damage. Where the scavenging process converts excess ROS into safer molecules, and this process is a safe alternative to the classical utilization of anti-inflammatory medications, which may harm several healthy organs. Nanomaterials can scavenge reactive oxygen species (ROS), thereby mitigating inflammation and oxidative-stress–induced tissue damage. By converting excess ROS into innocuous molecules, they represent a potentially safer alternative to conventional anti-inflammatory pharmacotherapy, which often risks collateral injury to healthy organs (Liu et al., 2023).

### 2.5 Structural support

MNPs like silver, zinc oxide, and copper oxide nanoparticles are usually integrated into the matrices of polymers, including chitosan, calcium alginate, and polyvinyl alcohol. The matrices enhance the stability of nanoparticles, which improve their mechanical properties, leading to a more effective and durable wound patch (Xu et al., 2023; Sarwar et al., 2022; Mosallanezhad et al., 2022; Balakrishnan et al., 2020). For example, using zinc oxide nanoparticles in nanofibers enhances their tensile strength and strain at breaking, enhancing the wound dressings’ adaptability and durability (Mosallanezhad et al., 2022). The mechanical properties of the polymers are enhanced by the uniform distribution of MNPs within their matrices. That is because well-dispersed nanoparticles assist in maintaining the matrices’ structural integrity (Zaragoza et al., 2018). Also, the incorporation of MNPs within polymer matrices reduces the existing interfacial defects, which is an important factor for enhancing the mechanical properties (Zou et al., 2022).

Integrating MNPs into nanofibers or hydrogels helps maintain control over the release of metal ions, which will reduce the possible toxicity while retaining the antibacterial properties (Xu et al., 2023). This is because certain hydrogels limit the leakage of metal ions when the MNPs are confined in the hydrogel’s network. Although the MNPs are confined within the matrix, they can still generate ROS under visible light, which will maintain the effectiveness of their antibacterial properties (Xu et al., 2023). Also, altering the MNP concentration in the hydrogel and the cross-linking density of the hydrogels can strongly influence the release of metallic ions, which enables the release to targeted regions while minimizing/preventing the release to non-targeted areas (Hezaveh and Muhamad, 2012).

**FIGURE 2:**
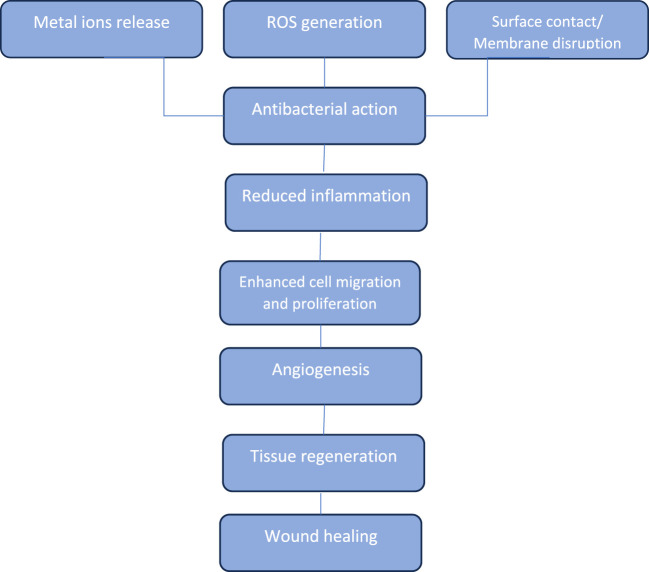
Flow chart of the integrated mechanistic pathways of MNP in wound healing.

Integrating MNPs into wound patches has been proven to be generally biocompatible and enhances the effectiveness of wound healing due to their ability to develop cell adhesion and propagation (Sarwar et al., 2022). The size and morphology of the nanoparticles have a crucial influence on their efficiency in such applications. For instance, nanoparticles with rod morphology indicated less cytotoxicity when compared to spherical nanoparticles. This suggests that the nanoparticles’ morphology may affect their interactions with human tissues and bacterial cells (Favi et al., 2015). The stability of the nanoparticles in the patches is also an important factor influencing their efficiency. To increase their stability, processes such as sonication can change their surface morphology to enhance their antibacterial effectiveness and structural properties (Ulasevich et al., 2020). A flowchart that outlines the common mechanistic pathways through which metallic nanomaterials contribute to wound healing is illustrated in Figure 2.

## 3 Metallic nanoparticles: key properties and applications in wound healing

### 3.1 Silver nanoparticles (Ag NPs)

**FIGURE 3:**
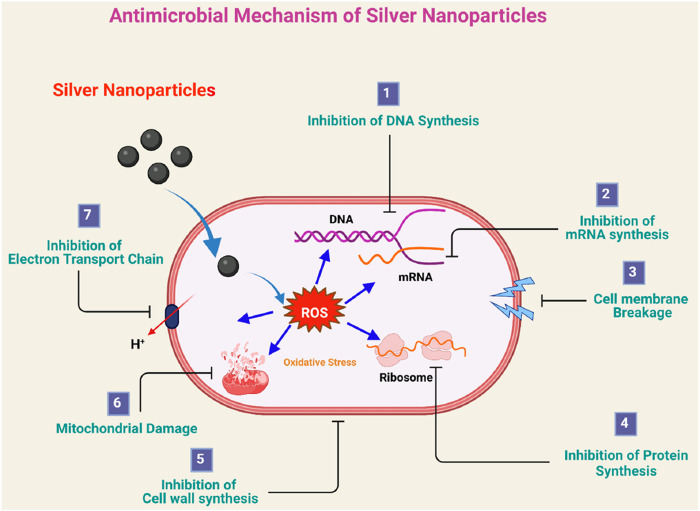
Antimicrobial mechanism of silver nanoparticles: (1) inhibition of DNA synthesis, (2) inhibition of mRNA synthesis, (3) cell membrane destruction and the leakage of the cell constituents, (4) inhibition of protein synthesis, (5) inhibition of cell-wall synthesis, (6) mitochondrial damage, and (7) inhibition of electron transport chain. Reproduced from Jain, A.S.; Pawar, P.S.; Sarkar, A.; Junnuthula, V.; Dyawanapelly, S. Bionanofactories for Green Synthesis of Silver Nanoparticles: Toward Antimicrobial Applications. Int. J. Mol. Sci. 2021, 22, 11993. https://doi.org/10.3390/ijms222111993. Licensed under CC BY 4.0. (https://creativecommons.org/licenses/by/4.0/).

Silver as a noble metal has been broadly utilized in treating wounds and preventing infections. The antibacterial effects of Ag NPs are highlighted by the bactericidal and inhibitory mechanisms (Figure 3). Ag NPs can eliminate bacteria by interacting with the Sulphur and phosphorus in their DNA (Sheikh-Oleslami et al., 2023). Also, if the Ag NPs are released into the wound, they can interact with the bacterial proteins that have a negative charge, causing a disruption to the cell walls and membranes of the bacteria. This effect occurs more significantly if the Ag NPs oxidize to Ag^+^ ions in acidic environments. Such damage will also cause damage to the mitochondria of the bacteria, which will lead to disruption of the bacterial cell respiration with out affecting the proliferation of normal human cells (Sheikh-Oleslami et al., 2023).

The existence of Ag NPs on the wound site promotes the generation of ROS, which induces oxidative stress on the bacterial cells to reduce their viability (Sheikh-Oleslami et al., 2023). Ag NPs promote accelerated, scar-free wound healing by aiding the growth of keratinocytes at the site. They also exhibit anti-inflammatory properties due to their ability to reduce cytokines and promote re-epithelialization (Sheikh-Oleslami et al., 2023). They are widely used in this field as shown in Table 2. The antibacterial effectiveness of Ag NPs is influenced by their size and concentration. However, if large quantities of Ag NPs are used, the healing process could be impaired due to the nanoparticles’ toxicity, which can damage human cells (Sheikh-Oleslami et al., 2023).

**TABLE 2:** Summary of several studies that utilize various MNPs in wound healing patches.

Nanoparticles	Patches material	*In vitro* test	*In vitro* findings	*In vivo* evaluation test	*In vivo* findings	References
Au NPs	Alginate hydrogel	Testing against *E. coli*, *S. aureus*, and *P. aeruginosa* bacterial strains, and cell biocompatibility testing	Killing more than 95% of the *P. aeruginosa* and *E. coli* bacterial strains and up to 60% of the *S. aureus*. There was more than 80% reduction in *P. aeruginosa* *E. coli* bacterial colonies and a 35.4% reduction in the *S. aureus* bacterial colonies. Cell viability >85%	Tests on S. aureus-infected wounds in rats	Rapid enhancement in healing with improved wound closure	Kaul et al. (2022)
Au NPs	Polyethyleneimine (PEI), polyethylene glycol (PEG), hexachloro cyclic triphosphoni trile (HCCP) hydrogel	Testing against *S. aureus* and methicillin-resistant *Staphylococcus aureus* (MRSA) bacterial strains and cell cytocompatibility testing	Good performance against the bacterial strains, specifically if the hydrogel is exposed to laser irradiation and good biocompatibility with minimal cytotoxicity	Tests on wounds in mice	Rapid wound healing without signs of adverse effects or toxicity when compared to control samples	Shang et al. (2022)
Au NPs	Chitin hydrogel	Testing against *E. coli* and *S. aureus* bacterial strains and cell cytotoxicity testing	Successful partial or full inhibition of the bacterial strains at certain concentrations of Au NPs. Cell survival of more than 80% for all groups after 48 h, depending on the Au NPs concentration	Tests on *S. aureus* infected wounds in mice	Good anti-inflammatory and antibacterial properties	Zhao et al. (2023)
Ag NPs	Gelatin hydrogel	Testing against *E. coli* and *S. aureus* bacterial strains and cell cytotoxicity testing	Inhibitory effects against both strains and good cell viability	Tests on second-degree burn wounds in rats	85% wound closure within 10 days, and skin layer structure was observed to be more developed compared to control samples	Li et al. (2022)
Ag NPs	Aloe vera-silk Fibroin composite	Testing against *E. coli* and *S. aureus* bacterial strains and cell cytotoxicity testing	Inhibitory zones in both bacterial strains with around 13.9 mm and 10.6 mm inhibition against *E. coli* and *S. aureus* strains, respectively, and good promotion of cell proliferation due to good biocompatibility	Tests on wounds in rats and biocompatibility test	Acceleration of healing, good antibacterial properties, acceptable biocompatibility, and promotion of cell proliferation compared to control samples	Liu et al. (2021)
Ag NPs	Chitosan hydrogel	Testing against *E. coli*, *S. aureus*, *P. aeruginosa*, K. pneumonia, and B. cereus bacterial strains and cell cytotoxicity testing	Inhibitory effects against all strains with inhibition zones in the range of around 11.52–21.47 mm and no toxicity for certain concentrations for 24 h	Tests on *P. aeruginosa* infected wounds in rats	Rapid healing and enhanced scar appearance and connective tissue production, in addition to lower bacterial counts when compared to control samples	Bharathi et al. (2021)
Ag NPs	PPCA	Testing against *E. coli* and *S. aureus* bacterial strains and cell cytotoxicity testing	Inhibitory effects against both strains and promotion of cell proliferation due to good cell viability and low toxicity	Tests on wounds in diabetic mice	97% wound closure within 14 days, while control patches achieved only 81% closure; notable inflammatory reduction and prominent angiogenesis regeneration	Lin et al. (2023)
Ag NPs	PF127 polymer	Testing against *E. coli*, *S. aureus*, *P. aeruginosa*, and B. cereus bacterial strains and cytotoxicity testing (fruit fly eggs)	Inhibitory effects against all strains with inhibition zones in the range 10.3–18.7 mm and no significant cytotoxic effect were observed on the fly eggs	Tests on wounds in mice	Wound closure is dependent on the Ag NPs concentration, and the Ag NPs patches showed more effective wound closure compared to control samples, and no skin irritation was observed during or after the testing	Chinnasamy et al. (2021)
Zn NPs (Zinc sulphide nanoparticles)	Gelatin methacryloyl hydrogel	Cytocompatibility testing	Cell viability remained close to 100% in 1, 3 and 5 days, showing no cytotoxic effect; proliferative ability is not influenced, showing good cytocompatibility	Tests on wounds in rats	Almost full wound closure by day 15, which shows accelerated and enhanced wound healing when compared to control samples that achieved around 70% wound closure by day 15	Zheng and Jin (2022)
Cu NPs (copper N-doped carbon dots with graphene oxide nanosheets)	Chitosan hydrogel	Testing against *E. coli* and *S. aureus* bacterial strains and cell cytotoxicity testing; cytotoxicity of Cu tested through MTT assay	Good antibacterial properties with a killing rate of 98.40% and 96.5% in *E. coli* and *S. aureus* bacterial strains, respectively. Exhibited less cytotoxicity than the control hydrogel. High cytotoxicity was observed when the hydrogel was exposed to laser radiation (54.6% cell viability), but later showed good cell viability (88.4%) after 1 week of culturing	Tests on skin wounds in mice	Enhanced wound healing with 15% more wound closure than control samples within 7 days. No significant cytotoxicity effects were observed, which suggests good biocompatibility	Wang et al. (2021)
Cu NPs	Polyacrylamide hydrogel	Testing against *E. coli* bacterial strain and cell biocompatibility testing	Reduction in the bacterial viability from 26.8% to 5.1% when increasing the raction of VBzTHPC from 2 to 10 mol%. Cell viability >85%	Tests on *E. coli* infected wounds in mice	Reduced inflammation through the generation and removal of collagen. Killed bacteria and accelerated the healing process compared to control samples	Guo et al. (2022)
Cu NPs (Copper sulfide nanoparticles)	3- (tri methoxysilyl) propyl methacrylate and mesoporous silica hydrogel	Testing against *S. aureus* and *E. coli* bacterial strains and cytotoxicity testing	Antibacterial effectiveness of 99.94% and 99.80% against *E. coli* and *S. aureus* bacterial strains, respectively, within 10 min of near-infrared light irradiation exposure; Cytotoxicity is dependent on the concentration and exposure time to NPs. Hydrogels with smaller concentrations of NPs do not exhibit appreciable negative effects on the cell viability, demonstrating the good biocompatibility	Tests on wounds in rats	The ability to kill the bacteria while advancing the healing process; no major organs are affected during a 14-day treatment	Li et al. (2018)
Cu NPs	Methacrylate-modified gelatin	Testing against *E. coli* and *S. aureus* bacterial strains and cell biocompatibility testing	Up to 91.2% and 90.8% of E. coil and *S. aureus* bacterial viabilities, respectively, when utilizing 8 mM of Cu NPs in the hydrogel and incubation for 6 h. No significant effects on cell proliferation and the measured inflammatory gene expression	Tests on *S. aureus* infected wounds in rats	Improved wound closure of 95.1% compared to the control samples, which achieved a 79.3% wound closure	Tao et al. (2019)
ZnO NPs	Chitosan hydrogel and poly (vinyl alchohol)	Testing against *E. coli* and *S. aureus* bacterial strains for both functionalized and non-functionalized hydrogels and cell biocompatibility testing	Good antibacterial properties with inhibition zones in the ranges of 34–39 nm and 33–38 nm when tested against *S. aureus* and *E. coli* bacterial strains, respectively; ZnO-based hydrogels (non-functionalized and heparin functionalized showed good contact with the cells	Tests on wounds in rats	Faster wound closure rate and reduced collagen deposition when compared to other samples	Khorasani et al. (2021)
TiO_2_ NPs	Gellan gum	Testing against Gram-negative and Gram-positive bacterial strains	The samples exhibited good antibacterial properties against both types of strains	—	—	Razali et al. (2020)
CuO NPs	Sodium carboxymethylated starch hydrogel	Testing against Gram-negative and Gram-positive bacterial strains and cell cytotoxicity testing	Good inhibition of both types of strains with an inhibition zone between 20 and 32 mm. High toxicity at high concentrations of CuO NPs is observed, possibly due to excessive ROS generation	Tests on wounds in rats	Enhanced the time frame of wound healing compared to control samples	Abdollahi et al. (2021)
Mg (OH)_2_ NPs	Carboxymethyl cellulose hydrogel	Cell viability, blood compatibility, and antibacterial testing against P. aeruginosa strain	The viability of cells exposed to the NPs-loaded scaffold had a viability of 84.5% after 7 days, while the control (untreated) group had a viability of 95.7%. The scaffold caused 8.3% red blood cell hemolysis. The scaffold reduced the bacterial proliferation and biofilm formation.	Tests on wounds in mice	Mice treated with the NPs-loaded scaffold achieved a wound closure of 82.29% while the wound closure in controls was 75.63%	Eivazzadeh-Keihan et al. (2021)
CeO_2_ NPs	Poly (3-hydroxybutyrate-co-3 -hydroxy valerate)	Cell viability, adhesion, and wound healing (scratch assay)	The membranes demonstrated a cell viability of >90% and improved adhesion, indicating high cytocompatibility. Also, the membranes achieved accelerated (>60%) wound closure compared to controls (40-60%) within 20 hours	Tests on wounds in diabetic rat model	The NPs-loaded membranes improved cell infiltration and granulation tissue formation	Augustine et al. (2020)
CeO_2_ NPs	Polyvinyl alcohol/Chitosanhydrogel	Testing against MRSA and *E. coli*​ bacterial strains and cell cytocompatibility testing	Good antibacterial properties against MRSA strain but no significant effect against the *E. coli* strain. NPs-loaded hydrogels indicated no significant toxicity after 5 days	—	—	Kalantari et al. (2020)
ZnO NPs and Ag NPs	Carboxymethyl cellulose hydrogel	Testing against *E. coli* and *S. aureus* bacterial strains and cell cytotoxicity testing	Bacteria killing rate of 95.95% and 98.49% for the *E. coli* and *S. aureus* bacterial strains after 20 min of normal light exposure. Inhibition zones were visible for the nanocomposite hydrogels, while the control samples had no significant inhibition zones. Cytotoxicity was observed for Ag NPs hydrogels. However, when combined with ZnO NPs, the cytotoxicity was reduced	Tests on *S. aureus* infected wounds in rats	The release of zinc and silver ions resulted in an increase in the number of white blood cells and neutrophils at least twice the amount when using control samples. This accelerated and enhanced the antibacterial properties	Mao et al. (2017)
Ag NPs and Cu NPs	Chitosan	Testing against *E. coli* and *S. aureus* bacterial strains and cell biocompatibility testing	Antibacterial activity against both types of strains and inhibition zones was observed after 24 h of testing. Biocompatibility showed good results for small concentrations of nanoparticles	Tests on *S. aureus* infected wounds in type 1 diabetic and non-diabetic rats	Acceleration of the healing process by the NPs-loaded hydrogel and major wound closure within 14 days	Liu et al. (2022)

In the following sections we discuss the silver nanoparticle-based formulations, their antibacterial mechanisms, wound healing efficiency, and their feasibility for clinical applications.

Singla et al. utilized the Ag NPs combined with bamboo cellulose nanocrystals (CNS) to fabricate a nanocomposite hydrogel wound dressing utilizing an *in situ* single-vessel methodology. The authors attributed this performance to the synergistic interplay between the structural support afforded by bamboo-derived cellulose nanocrystals and the broad-spectrum bactericidal effect of embedded silver nanoparticles, which together sustain a moist microenvironment favorable to tissue regeneration. They synthesized the Ag NPs by reducing a silver nitrate solution with a leaf extract of Syzygium cumini (Singla et al., 2017). A similar green methodology was executed by Ahsan et al. for the synthesis of Ag NPs utilizing cabbage extract. They incorporated the nanoparticles into a hydrogel of polyvinyl alcohol (PVA) to make the hydrogel applicable as a wound patch (Ahsan and Farooq, 2019). Researchers from Chungnam National University incorporated Ag NPs into thermo-responsive methylcellulose (MC) hydrogel to fabricate a dressing for burn wound healing that possesses enhanced wound regeneration and antibacterial properties *via* the release of ions mechanism (Kim et al., 2018).

**FIGURE 4:**
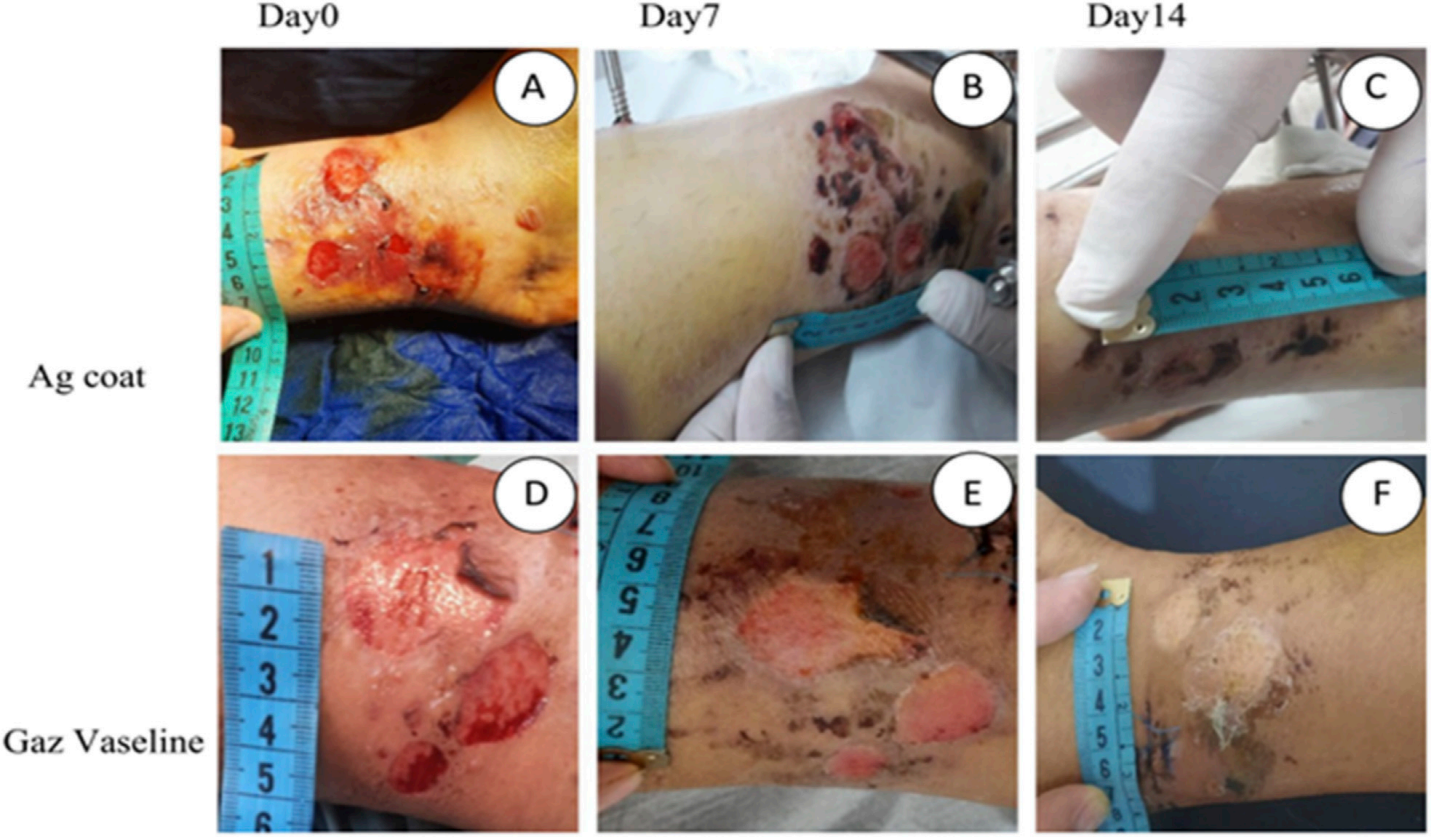
Macroscopic view of the blisters and healing process based on dressing type. **(A, D)** Blisters in the frst day, **(B)** day 7 in Ag coat group, **(C)** day 14 in Ag coat group, **(E)** day 7 in Gaz Vaseline group, **(F)** day 14 in Gaz Vaseline group) (Teimouri and sadat Lalehzar, 2023). Reproduced from Teimouri, M.; Lalehzar, S. Evaluation of the therapeutic effect of dressing containing Silver (Ag coat) in the process of healing skin blisters caused by limb fractures: a clinical trial study. BMC Surg. 2023, 23, 101. https://doi.org/10.1186/s12893-023-02012-8. Licensed under CC BY 4.0. (https://creativecommons.org/licenses/by/4.0/).

Along with the innovative approaches in hydrogels, the silver nanoparticle delivery have also been developed in most noval ways. The study by Singla et al. had integrated the synthesized Ag NPs into the CNCs matrix, and the composite was made in film and ointment forms to be utilized as a dressing. (Singla et al., 2017). According to Ahsan et al., they fabricated the patches using a freeze-thaw process, and then explained that such a process increases the hydrogel’s structural stability. They characterized the fabricated nanoparticles thoroughly, and they evaluated the capabilities of the patches in wound healing and bacteria reduction (Ahsan and Farooq, 2019). Teimouri and Lalehzar coated wound dressings with silver nanocrystals (Ag coat) to assess their effectiveness in healing the skin blisters that are caused by limb fractures when compared to the traditional Gaz Vaseline dressings, as seen in Figure 4 (Teimouri and sadat Lalehzar, 2023). Moreover, the patients experienced less pain while changing the Ag coat dressings and a reduced number of dressing changes compared to Gaz Vaseline dressings. This indicates that the Ag coat dressings are cost-efficient, time-efficient, and possess pain-relieving effects.

Silver nanoparticle coatings will exert their therapeutic effect primarily through the antibacterial mechanisms, including the metal ions release, ROS generation and the bacterial cell walls disruptions. Singla et al. explain that the nanocomposite hydrogel fabricated with bamboo cellulose nanocrystals (CNS) incorporating the silver nanoparticles, combines the antibacterial properties and structural support benefits of the CNCs and Ag NPs, maintaining a moist environment at the wound site that is crucial for effectively controlling bacterial infection and enhancing wound healing (Singla et al., 2017). Ahsan et al. performed *in vitro* testing of the patches by measuring the antibacterial activity against *S. aureus* and *Escherichia coli* bacterial strains, and their results showed that the patches had significant antibacterial activity against such strains (Ahsan and Farooq, 2019). Kim et al. assessed the performance of the hydrogels against *S. aureus*, *E. coli*, and *Klebsiella pneumoniae* bacterial strains. The results indicated that the hydrogel inhibited the growth of bacteria for all strains at a rate of 99%. They also found that 95% of the silver ions are released within the first 6 hours, which promotes rapid bacterial inhibition. The hydrogels exhibit strong antibacterial properties and enhance wound healing through structural support (Kim et al., 2018).

Along with the antimicrobial activity, the silver nanoparticles will help in wound healing by promoting collagen deposition, reducing inflammation, and tissue regeneration. The findings by Singla et al. in their *in vivo* experiments on mice revealed that hydrogels reduced inflammation, enhanced collagen content, and promoted rapid wound closure. They observed a full wound closure on the 14th day of the healing process. Benefits of the CNCs and Ag NPs along with the hydrogels help in maintaining a moist environment at wounds site which is crucial for the wound healing (Singla et al., 2017). A similar result was obtained for the study conducted by Ahsan et al. , whose results indicated that the Ag NPs patches facilitated fast, significant closure within 14 days, when compared to the wounds treated with control samples (without nanoparticles) and samples treated with standard healing drugs. Also, wounds treated with Ag NPs patches illustrated reduced inflammation and increased collagen formation compared to other samples. The Ag NPs patches illustrated rapid wound healing and strong antibacterial activity; in addition to that, the freeze-thaw methodology enhanced the structural stability of the hydrogels, which makes them potential options in wound healing applications (Ahsan and Farooq, 2019). Teimouri and Lalehzar, in their study in which they coated wound dressings with silver nanocrystals, showed that the participants were randomly given Ag-coated dressings or Gaz Vaseline dressings. To evaluate the performance effectively, the blisters were monitored over 0, 7, and 14 days and assessed based on several factors, including pain scoring and wound size measurement. The Ag coat dressings exhibited faster and more effective healing than the Gaz Vaseline dressings. Also, the Ag coat dressings reduced the wound area and inflammation considerably compared to Gaz Vaseline dressings; by day 14, the patients had a noticeable decrease in the wound area (Teimouri and sadat Lalehzar, 2023). Kim et al. performed an *in vivo* performance assessment by applying the dressing to burn wounds in rats These tests showed that the nanocomposite promoted the formation of more collagen and enabled the significant generation of tissues when compared to untreated groups (Kim et al., 2018).

Despite the promising outcomes, there are still limitations in translating the laboratory outcomes to a large scale level. Preserving uniform nanoparticle dispersion may benefit from further optimization (Singla et al., 2017). The safety of the green methodology, the long-term effects of the patches, and their effectiveness in humans may benefit from being further investigated (Ahsan and Farooq, 2019). The fast release of silver ions may decrease the effectiveness of the patches over time, and hence, further studies that optimize the release rate should be conducted (Kim et al., 2018).

### 3.2 Gold nanoparticles (Au NPs)

Au NPs, as noble metals are utilized in wound healing applications due to their biocompatibility and antioxidant properties. The antibacterial effectiveness of Au NPs on their own (i.e., without merging with other materials) is usually present but with low activity, unlike Ag NPs (Sheikh-Oleslami et al., 2023). Au NPs can eliminate bacteria by changing their membrane potential as the nanoparticles enter the cells. This breaks down the energy metabolism, leading to eventual cell death (Sheikh-Oleslami et al., 2023). Also, Au NPs promote the generation of ROS, which induces oxidative stress on the bacterial cells, reducing their viability (Sheikh-Oleslami et al., 2023). The antibacterial effectiveness of Au NPs increases with the increase in their surface area (i.e., diameter decrease) (Sheikh-Oleslami et al., 2023). The antioxidant properties of Au NPs are due to their ability to bind with free radicals such as hydroxyl (OH^−^). Moreover, Au NPs boost collagen expression, reduce cell death, and promote cell growth and new blood vessel formation, which all promote the acceleration of the healing process (Sheikh-Oleslami et al., 2023).

Akturk et al. incorporated Au NPs in collagen-based nanocomposite scaffolds to be used as a wound-healing patch (Akturk et al., 2016). The Au NPs that were used had a size greater than 20 nm and were integrated into the collagen scaffolds. The scaffolds were then cross-linked with glutaraldehyde to improve their resistance to degradation and enhance their mechanical stability. The results of the study indicated that the cross-linked Au NP-collagen scaffolds have higher stability, higher tensile strength, and reduced degradation rate compared to the Au NP-collagen scaffolds that are not cross-linked. In the study conducted by Chen et al. they used a mixture of Au NPs with a supramolecular peptide-based hydrogel (NapFFY) to fabricate a composite wound dressing for wound healing applications (Chen et al., 2023). The role of nanoparticles is to provide anti-inflammatory and antibacterial functions, while the hydrogel acts as a self-assembling biocompatible scaffold. At the same time Mahmoud et al. used the poloxamer 407 hydrogel to enhance wound healing by incorporating Au NPs in the hydrogel (Mahmoud et al., 2019). They synthesized Au NPs of different morphologies and surface charges and incorporated them into the hydrogel dressing. Au NPs with distinct morphologies and surface charges were synthesized and integrated into the hydrogel matrix to enhance its functional properties.

The findings of Mahmoud et al. showed that the added Au NPs inhibited the growth of *S. aureus* and *Pseudomonas aeruginosa* bacterial strains, and promoted a regenerative wound environment, as the inflammatory markers were reduced (Mahmoud et al., 2019). The *in vitro* assessments made by Chen et al. indicated that the Au NPs-loaded hydrogel inhibited the growth of *S. aureus* and *E. coli* bacterial strains significantly, achieving an efficacy of nearly 80% while maintaining low hemolysis rates and excellent cytocompatibility (Chen et al., 2023).

Integrating Au NPs into collagen scaffolds improved the structural and biological properties of the scaffolds by accelerating the wound healing process, reducing inflammation, enhancing mechanical properties, and promoting cell proliferation and attachment. In the *in vivo* testing done by Akturk et al. involved the observation of wound healing on a rat skin wound, while the *in vitro* testing involved the evaluation of cell viability after exposure to different concentrations of Au NPs. (Akturk et al., 2016). The results of the *in vivo* tests indicated that the scaffolds with Au NPs enhanced tissue formation, stimulated mild inflammatory responses, and achieved greater wound closure compared to untreated scaffolds (without Au NPs). On the 14th day of the healing process, the untreated scaffolds achieved 45% wound closure, while the Au NPs scaffolds achieved 69%. The *in vitro* testing results indicated that utilizing low concentrations of Au NPs resulted in non-toxic scaffolds that have high biocompatibility and show no critical effects on the cell viability (Akturk et al., 2016). Along with these advances, the research by Mahmoud et al. also applied the dressings on rat wounds for 21 days, and the healing effectiveness was evaluated using several factors, including wound closure, inflammatory gene expression, and antibacterial effects. The results indicated that certain hydrogels, such as cationic Au NPs hydrogels, accelerated the healing and wound closure, where the wound was almost closed in 14 days. The hydrogel promotes fast healing with minimal scarring and reduces infection while maintaining the release of Au NPs to provide tissue repair and infection reduction. (Mahmoud et al., 2019). At the same time the *in vivo* experiments by Chen et al., which were carried out on full-thickness skin incision models in rats, indicated that the groups treated with the Au NPs-loaded hydrogel exhibited minimal inflammation, extensive tissue regeneration, and faster wound closure compared to the groups treated with hydrogel or Au NPs on their own. Histological and Masson staining showed that the Au NPs-hydrogel treated group had faster angiogenesis and collagen deposition, which indicated that it healed better. These findings suggest that the Au NPs-loaded hydrogel is successful in providing wound management (Chen et al., 2023).

However, the authors suggest exploring the effects of using higher Au NP concentrations on the effectiveness of the scaffolds in wound healing, and the need for more *in vivo* studies to indicate any risks that might be associated with Au NP usage remains (Akturk et al., 2016). Mahmoud et al. study’s drawbacks are that some of the tested hydrogels did not exhibit such a superior effect. Also, small concentrations of Au NPs were observed in the rat organs. Hence, the authors recommend that further studies regarding long-term safety must be conducted (Mahmoud et al., 2019).

### 3.3 Zinc oxide nanoparticles (ZnO NPs)

Zn and ZnO NPs are cost-effective, biocompatible, and non-toxic, and possess antibacterial and anti-inflammatory properties, which makes them useful for wound healing (Sheikh-Oleslami et al., 2023; Aminzai and Patan, 2024). Their antibacterial properties mainly rely on their ability to induce oxidative stress and rupture the bacterial cell membranes (Sheikh-Oleslami et al., 2023). When the nanoparticles release zinc ions into the wound site, the activity of keratin-producing cells increases, which accelerates the healing process (Aminzai and Patan, 2024). Their antibacterial effect increases with increasing surface area, while the ion release aids in forming new blood vessels for tissue repair and healing (Aminzai and Patan, 2024).

Researchers have incorporated ZnO NPs into Polyvinyl Alcohol (PVA)/chitosan hydrogel to enhance the hydrogel’s biocompatibility and antibacterial properties. The nanocomposite hydrogels were prepared using the freeze-thaw methodology to achieve high porosity and enhance their biocompatibility (Khorasani et al., 2018). Manuja et al. developed a nanocomposite wound dressing made of alginate, gum acacia, and ZnO NPs to accelerate the wound healing process, with the main target of enhancing skin regeneration in excised wounds in rabbits (Manuja et al., 2020). The nanoparticles were synthesized using a hydrothermal method and then incorporated into the composite. They characterized the elemental composition of the nanocomposite in addition to the morphology and size of ZnO NPs. As the detection of NO indicates the presence of oxidative stress they assessed the nanocomposite performance by measuring the wound closure, collagen content, and the release of nitric oxide (NO) (Manuja et al., 2020). ZnO NPs synthesized by Blinov et al. were stabilized using biopolymers that include sodium carboxymethyl cellulose (Na-CMC) and sodium alginate to enhance their biocompatibility and dispersion stability (Blinov et al., 2023). The stabilized ZnO NPs were integrated into composite gels designed for wound-healing and characterized using UV–Vis spectroscopy, Scanning Electron Microscopy, X-Ray Diffraction, and Fourier Transform Infrared Spectroscopy to validate the formation of nanoparticles and the polymer interactions.

Nanocomposite hydrogels were characterized thoroughly and evaluated for their antibacterial effectiveness against *Staphylococcus aureus* and *Escherichia coli* bacterial strains. The results indicated that nanocomposite hydrogels have a strong effect on both bacterial strains when compared to control samples (without ZnO NPs), showing a 70% inhibition against both bacterial strains. The biocompatibility testing of the hydrogels indicated low cytotoxicity, given that the cell viability after 48 h was 80%. The hydrogels enhance healing by continuously and slowly releasing zinc ions that offer antibacterial properties to the wound sites (Khorasani et al., 2018) The nanocomposites that are stabilized using biopolymers exhibited considerable enhancement in the antibacterial effects and less oxidative stress, identified by NO release, when compared to other types of treatments. The nanocomposite accelerated the wound healing process, reduced inflammation, exhibited biocompatible properties, and showed antibacterial effects, which reduced infection (Manuja et al., 2020).

Researchers have reported that the addition of nanoparticles enhanced the swelling capacity, water vapor permeability, and porosity of the hydrogels, which contributes to the enhanced healing process. The hydrogels enhance healing by continuously and slowly releasing zinc ions that offer antibacterial properties to the wound sites. The mechanical and permeability properties of the hydrogels were enhanced by the incorporation of ZnO NPs, which helped to maintain a moist wound site (Khorasani et al., 2018). The nanocomposite developed by Manuja et al., which were developed specifically for the wound healing caused the wounds in rabbits to close completely within 14 days, and the wound site had high collagen deposition and tissue formation. The increased collagen formation and calcium deposition are vital factors for tissue repair and strengthening. While the matrix provided environmental conditions that supported wound healing, such as a moist area (Manuja et al., 2020). Blinov et al. assessed the performance of the gels in enhancing wound-healing using a full-thickness skin wound model in rats, and the results indicated that the nanoparticle-loaded gels accelerated wound closure significantly compared to the control groups. The histological analysis concluded that the nanoparticle-loaded gels exhibit effective anti-inflammatory and regenerative activities due to the observation of granulation tissue formation, enhanced epithelialization, and decreased inflammatory cell infiltration in the nanoparticle-treated group (Blinov et al., 2023).

The authors explain that the antibacterial properties are attributed to ZNO NPs, which are associated with a reduction in inflammation (Manuja et al., 2020). However they highlighted that optimization of ZnO NPs-loaded hydrogel formulation is important to achieve desirable dressing properties (Khorasani et al., 2018).

**FIGURE 5:**
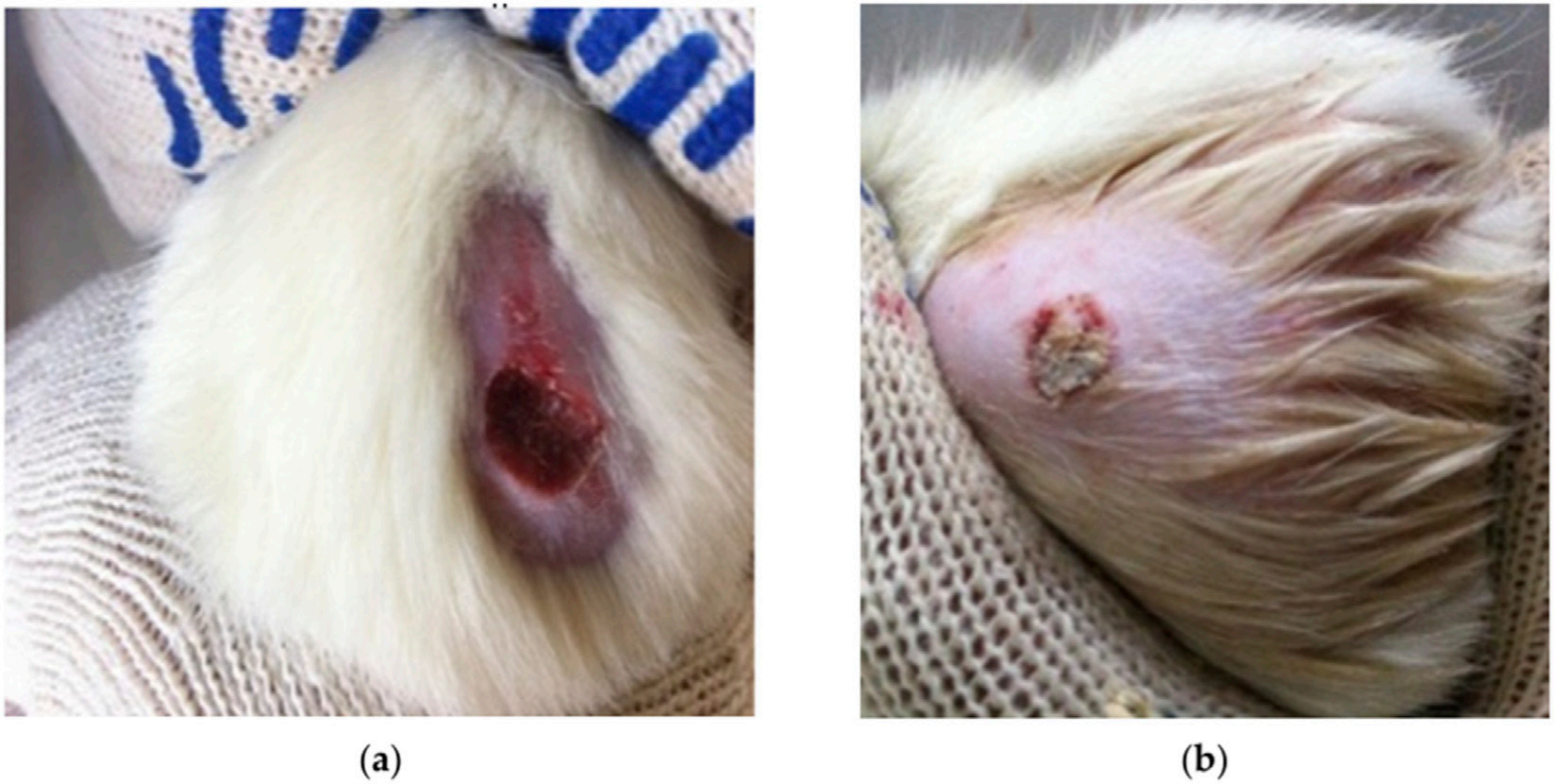
Photos of burn wounds of laboratory animals of group 1: **(a)** 3rd day of the experiment, **(b)** 10th day of the experiment (Blinov et al., 2023). Reproduced from Blinov, A.V.; Kachanov, M.D.; Gvozdenko, A.A.; Politaeva, N.V.; Golikova, M.V.; Smirnova, M.A.; Kudryashova, E.V. Synthesis and Characterization of Zinc Oxide Nanoparticles Stabilized with Biopolymers for Application in Wound-Healing Mixed Gels. Gels 2023, 9 (1), 57. https://doi.org/10.3390/gels9010057. Licensed under CC BY 4.0. (https://creativecommons.org/licenses/by/4.0/).

Figure 5 shows the photos of burn wounds after 3 and 10 days of the experiment.

### 3.4 Titanium dioxide nanoparticles (TiO_2_ NPs)

TiO_2_ NPs as transition metal oxides have high potential in being used in wound patches as they have excellent properties that are beneficial for wound healing, including anti-inflammatory and antibacterial activities, biocompatibility, and hydrophilicity (Dam et al., 2023). When TiO_2_ NPs are exposed to UV light, they generate ROS, which causes oxidative stresses that damage the cell walls and membranes of the bacteria and result in bacterial death (Younis et al., 2023; Priyadarshini et al., 2020). Also, when exposed to UV light, TiO_2_ NPs have shown enhanced photocatalytic activity, enhancing the effectiveness against bacteria through heightened ROS generation. Additionally, TiO_2_ NPs have shown enhanced photocatalytic activity, enhancing the effectiveness against bacteria through heightened ROS generation upon exposure to UV light (Younis et al., 2023; Lu B. et al., 2023). Furthermore, TiO_2_ NPs can interact with the cell membranes of bacteria, causing them to depolarize and lose their integrity, which makes the cell more permeable and causes their contents to leak, leading to eventual death (Sohm et al., 2015).

Khalid et al. fabricated nanocomposite dressings by integrating TiO2 NPs into bacterial cellulose (BC) sheets (Khalid et al., 2017). They characterized the composite’s properties and structure thoroughly to confirm the successful integration of TiO2 NPs (Khalid et al., 2017). A completely different fabrication of green method was done by Rahamanpour et al., who integrated TiO2 NPs and aluminum oxide nanoparticles (Al2O3 NPs) into a chitosan matrix to form a nanocomposite (Rahmanpour et al., 2022). They used oak fruit extract to reduce the metals into nanoparticles and stabilize the nanoparticles after their formation. The nanocomposite was prepared in an ointment form and tested on infected wounds in mice. In their *in vitro* assessments, they evaluated the effectiveness of the nanocomposite against *Staphylococcus aureus* and *Escherichia coli* bacterial strains.

The antibacterial activity of the nanocomposite was tested by the researchers using the agar disc diffusion methodology against *S. aureus* and *E. coli* bacterial strains (Khalid et al., 2017). The results of the tests indicated that the nanocomposite has significant antibacterial properties, where it was able to inhibit 83% of the *S. aureus* bacterial strain and 81% of the *E. coli* bacterial strain (Khalid et al., 2017) (Rahmanpour et al., 2022). The infected wounds in mice are treated as a test using the nanocomposites prepared in an ointment form. Their results indicated that the nanocomposite demonstrates significant antibacterial properties and accelerates wound closure when compared to control samples (without nanoparticles). The mice that were treated with the nanocomposite showed less swelling and increased cell proliferation compared to the ones treated with control samples. Also, the nanocomposite regulated the gene expression by promoting growth factors associated with wound healing and reducing inflammatory markers (Rahmanpour et al., 2022).

TiO2 NPs had been explored as a great remedy for the wound healing. The study by Khalid et al. who fabricated nanocomposite dressings by integrating TiO_2_ NPs into bacterial cellulose (BC) sheets, included an *in vivo* test on a mouse model with induced burn wounds, where they monitored the wound closure and tissue regeneration progress (Khalid et al., 2017). The *in vivo* testing indicated that the nanocomposite facilitated fast wound closure compared to control samples (without TiO2 NPs), as the closure of 71% was achieved by day 15 of the healing process. They also observed the generation and growth of healthy healing tissues, while incomplete healing and inflammation were observed with the control samples. The hydrophilic properties of the BC sheets made the wound site optimal for healing, and the properties of TiO2 NPs provided antibacterial effectiveness and tissue regeneration, which makes the nanocomposite a potential candidate for utilization in wound care (Khalid et al., 2017). The study by Rahamanpour et al. included an *in vivo* assessment where they monitored the wound closure, proliferation, and inflammatory expressions over 3, 7, and 12 days (Rahmanpour et al., 2022).

**FIGURE 6:**
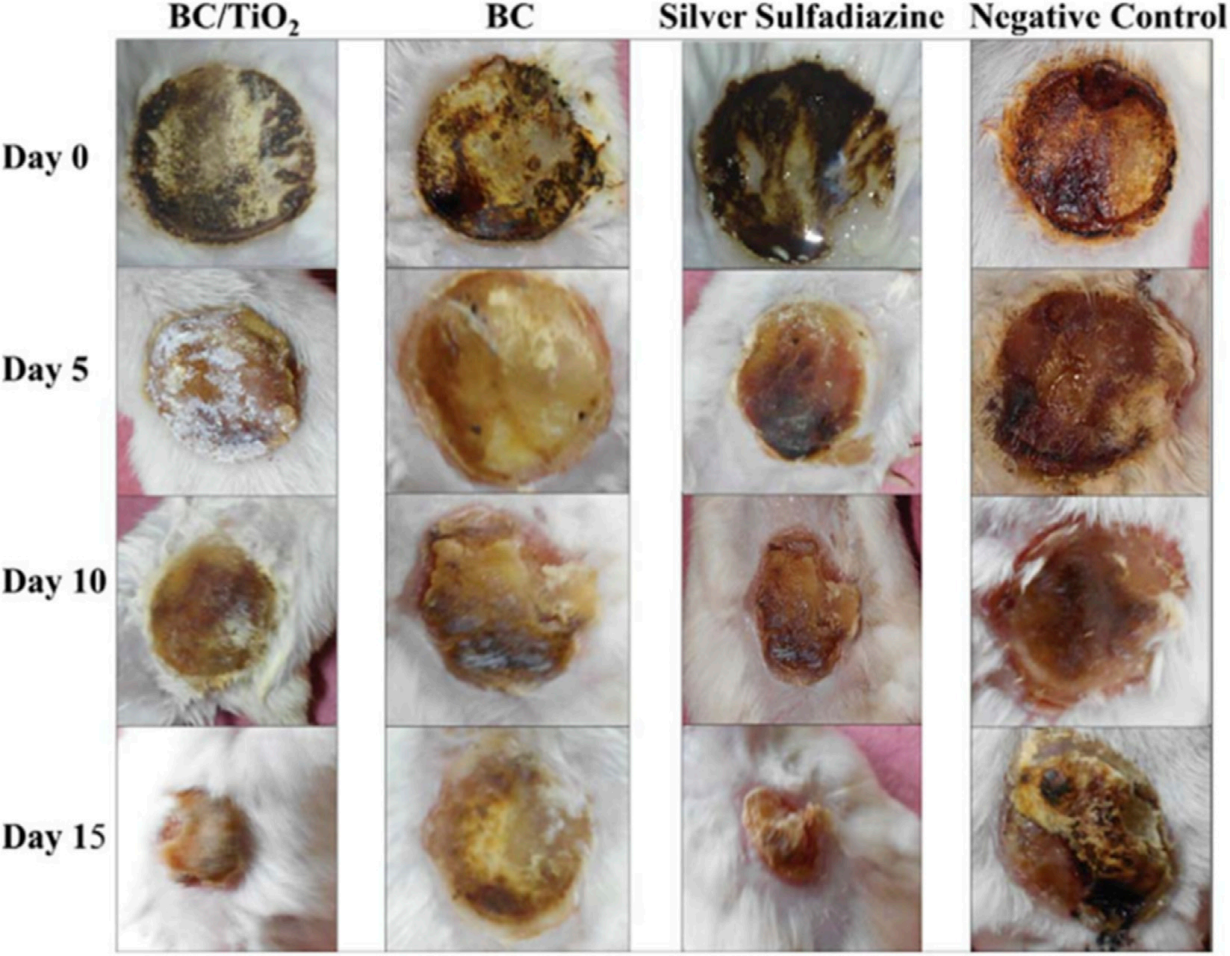
Wound photographs of all treated groups including BC–TiO_2_, BC, silver sulfadiazine and negative control groups on different treatment days. The prominent healing effects of BC–TiO_2_ can be seen in comparison to simple BC group (Khalid et al., 2017). Reproduced from Khalid, A.; Ullah, H.; Ul-Islam, M.; et al. Bacterial cellulose–TiO_2_ nanocomposites promote healing and tissue regeneration in burn mice model. RSC Adv. 2017, 7, 47662–47668. https://doi.org/10.1039/C7RA06699F. Licensed under CC BY 3.0 Unported. (https://creativecommons.org/licenses/by/3.0/).

Figure 6 presents the wound photographs at day 0, day 5, day 10, and day 15 from starting the experiment.

### 3.5 Copper nanoparticles (Cu NPs) and copper oxide nanoparticles (CuO NPs)

Cu and CuO NPs as transition metal oxides are known to be used as antibacterial agents that accelerate the wound healing process. Such nanoparticles engage in the production of essential growth proteins, which are crucial for wound healing, through cell division processes (Aminzai and Patan, 2024). Although they are useful in bacteria reduction, studies have shown that these types of nanoparticles are toxic compared to other MNPs. Usually, materials such as Folic acid are used with such nanoparticles to slow the ions release, which decreases the toxicity and enables cells to be mobile, and finally, wound healing is promoted (Aminzai and Patan, 2024).

Arendsen et al. integrated copper nanoparticles in wound dressings to assess their effectiveness in healing wounds in women who underwent cesarean sections (Arendsen et al., 2020). They applied two types of dressings to different patients: with and without (control) copper-oxide nanoparticles. The main goal was to assess the dressing’s effectiveness in reducing surgical site infections within 30 days of the surgical operation. Their results indicated that the infections at the wound site were 38.7% less for the copper-oxide nanoparticles dressings compared to the control dressings. Also, the severe cases of infections in women who used the nanoparticle dressings were 2.5%, while it was 12.7% for patients who used the control samples. The nanoparticle dressings were able to achieve a significant reduction of infections at the wound site without the use of antibiotics. This makes such dressings useful in cases where patients are exposed to bacteria present in the hospitals or suffer from antibiotic resistance. This study has some limitations, such as the fact that the diagnosis of wound infection was based on the reliance on antibiotics and self-reported symptoms. Also, since this was a pilot study, further studies are needed (Arendsen et al., 2020).

### 3.6 Magnesium hydroxide nanoparticles (Mg (OH)_2_ NPs)

Mg (OH)_2_ NPs as hydroxides can exhibit antibacterial properties *via* various mechanisms. Mg (OH)_2_ NPs can kill bacterial cells by physically interacting with them, causing physical damage to their structure, which leads to their death (Nakamura et al., 2021). Also, Mg (OH)_2_ NPs can cause oxidative stress in the cells of bacteria, causing cell membrane leakage, which results in cell death (Xia et al., 2024). The effectiveness of the antibacterial activity of Mg (OH)_2_ NPs is influenced by their size, where nanoparticles of smaller size exhibit more significant antibacterial properties compared to large-sized nanoparticles (Nakamura et al., 2021).

Qu et al. developed a multinetwork hydrogel incorporating Mg(OH)_2_ NPs for use as an antibacterial wound dressing (Qu et al., 2022). The inclusion of Mg(OH)_2_ NPs aimed to enhance both the hydrogel’s antimicrobial efficacy and its mechanical robustness. To improve nanoparticle dispersibility, the surfaces of the synthesized Mg NPs were functionalized with ricinoleic acid. The hydrogel matrix included various components, notably chitosan. Chitosan and PEG was used for functionalizing Mg (OH)2 NPs synthesized utilizing a hydrothermal methodology to reduce aggregation and enhance their biocompatibility and stability. The nanoparticles were integrated into alginate gels, and their performance in assisting wound healing was assessed using *in vivo* excision wound models in *Gallus domesticus* and chorioallantoic membrane (CAM) assay. (Abbas et al., 2024).

Mg(OH)_2_ NPs were aimed to enhance both the hydrogel’s antimicrobial efficacy and its mechanical robustness. The hydrogel matrix that included chitosan demonstrated strong inhibitory effects against *Staphylococcus aureus* and achieved a 50% reduction in viability against *Escherichia coli*. The authors explain that the antibacterial effects are due to the release of magnesium ions that disrupt the bacterial cell membranes, which causes a reduced risk of infection (Qu et al., 2022). A similar but improved efficiency was seen in the study conducted by Abbas et al., where the chitosan-functionalized nanoparticles provided enhanced anti-biofilm and antibacterial activities against *S. aureus* and *E. coli* bacterial strains, with a biofilm inhibition of 66.2% (Abbas et al., 2024).

The *in vivo* studies performed using rat skin models to evaluate wound healing performance indicated that the hydrogel promoted faster wound healing compared to traditional dressings with reduced inflammation. The developed hydrogels exhibit strong antibacterial properties, biocompatibility, and mechanical properties, making them a potential option in wound healing care (Qu et al., 2022). Abbas et al. in their study, concluded that the nanoparticle-loaded gels exhibited the highest wound closure rates (96.04% by day 9) compared to the chitosan-coated (89.76%) and unfunctionalized Mg (OH)2 NPs (84.77%). The Hemolysis assay confirmed that the chitosan- and PEG-coated nanoparticles have improved blood compatibility compared to the unfunctionalized nanoparticles (Abbas et al., 2024).

The hydrogels developed by Qu et al. exhibits strong antibacterial properties, biocompatibility, and mechanical properties, making them a potential option in wound healing care. However, the authors assert that further studies that test long-term effectiveness and biocompatibility need to be conducted (Qu et al., 2022). During the histopathological analysis of hydrogels synthesized utilizing a hydrothermal methodology, there was no sign of toxicological effects on the heart or liver tissues, which indicates the safety of such materials for biomedical applications (Abbas et al., 2024).

**FIGURE 7:**
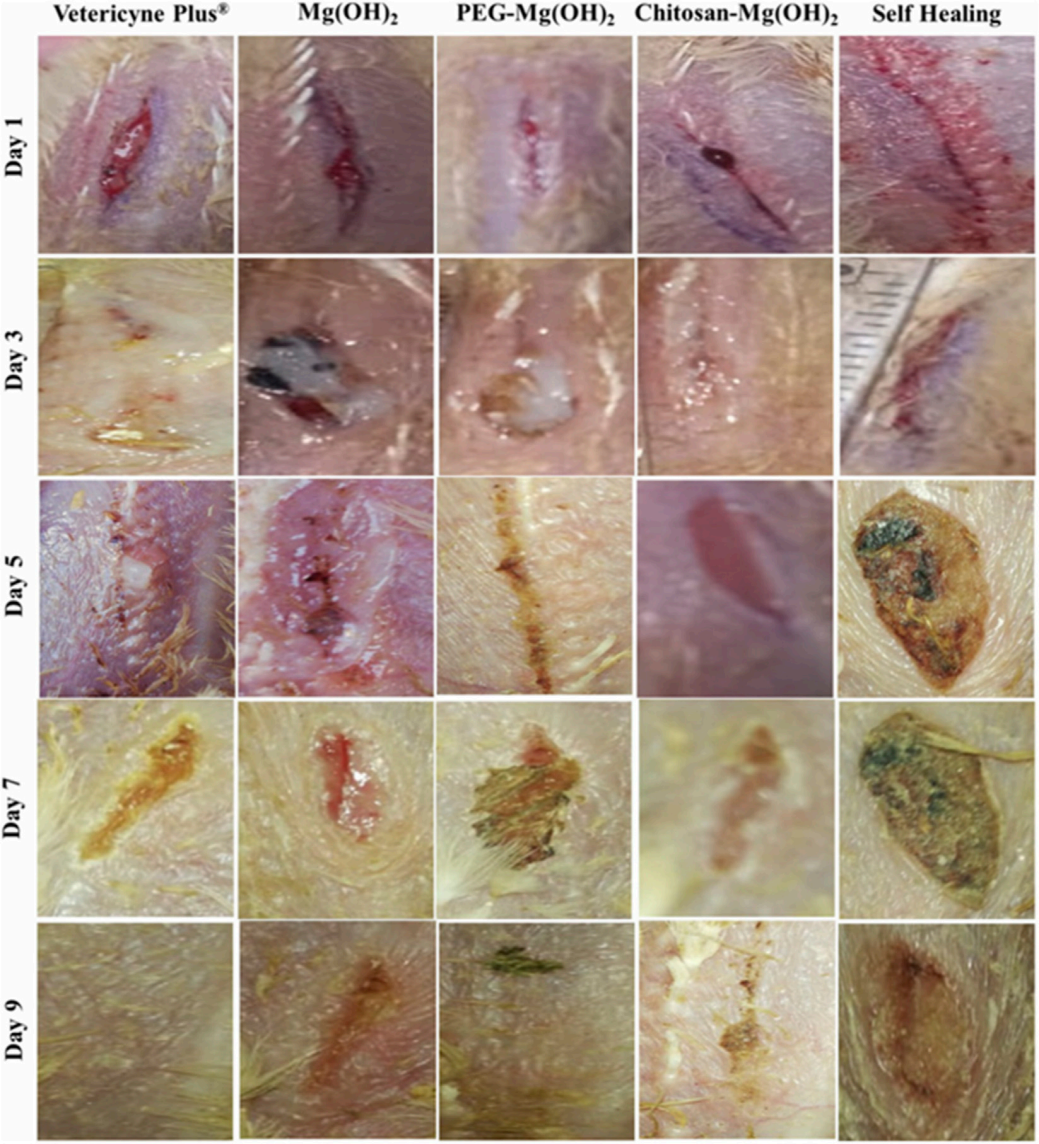
Photographic analysis of wound healing on the 1st, 3rd, 5th, 7th, and9th day after incision on *Gallus domesticus* (Abbas et al., 2024). Reproduced from Abbas, M.K.; Javed, Y.; Shad, N.A.; Rasool, N.; Shah, A.A.; Rashid, M.; Rafiq, M.; Abbas, S.R.; Haider, A.; Imran, M. Polymer coated magnesium hydroxide nanoparticles for enhanced wound healing. New J. Chem. 2024, 48, 17396–17409. https://doi.org/10.1039/D4NJ01909A. Licensed under CC BY 3.0 Unported. (https://creativecommons.org/licenses/by/3.0/).

Figure 7 illustrates the photographic analysis of wound healing after 1, 3, 5, 7, and 9 days from starting the experiment

### 3.7 Cerium oxide nanoparticles (CeO_2_ NPs)

CeO_2_ NPs are proven to have strong antibacterial properties that can be achieved through various mechanisms. CeO_2_ NPs can cause cleavage of bacterial DNA, which inhibits bacteria from replicating and causes bacterial cellular death (Yefimova et al., 2023). Also, CeO_2_ NPs can scavenge the nutrients from the environment of bacteria, which causes nutrient starvation and eventual bacterial death (Sargia et al., 2017). Furthermore, CeO_2_ NPs tend to generate ROS, which damages the cellular components of bacteria, leading to their death (Sargia et al., 2017). The effectiveness of CeO_2_ NPs in killing bacteria is highly influenced by the chemical and physical environment of the targeted region, as in certain conditions, CeO_2_ NPs were found to have no effect on bacteria (Shah et al., 2012).

Augustine et al. synthesized CeO_2_ NPs and integrated them into GeIMA hydrogel wound patches with the aim of using the free radical scavenging mechanism to enhance the healing of diabetic wounds, where oxidative stresses are high (Augustine et al., 2021). They characterized the patches for free radical scavenging and wound healing effectiveness. To assess the performance of the patches, they were applied to wounds in diabetic rats along the cell proliferation and healing process. The evaluation indicated that the nanoparticle-loaded patches (1%–4% CeO2 NPs) successfully scavenged free radicals, leading to reduced oxidative stress in the wound area. The patches with 1% CeO_2_ NPs had a significant effect on closing the wounds compared to the control (without CeO_2_ NPs) patches. Also, it was observed that the patches enhanced cell proliferation. The patches successfully reduced oxidative stress, accelerated wound healing, and supported cell viability in diabetic wounds. However, the authors explain that the control of CeO_2_ NPs concentration in the patches is important to ensure effectiveness in reducing oxidative stresses and to avoid potential toxicity (Augustine et al., 2021).

### 3.8 Multi-material nanoparticles

Researchers have combined the benefits of both ZnO NPs and CuO NPs by integrating them into a hydrogel composite made of gelatin, hyaluronic acid (HA), chondroitin sulfate (CS), and asiatic acid (Thanusha et al., 2018). They developed such hydrogel dressings with the aim of enhancing the healing of second-degree burn wounds. They prepared the composite by simply mixing the hydrogel materials with the nanoparticles. The hydrogel’s properties, such as biocompatibility, were characterized, and its ability to inhibit *Staphylococcus aureus* and *Escherichia coli* bacterial strains was assessed. Also, an *in vivo* study was conducted on rats with second-degree burns over a period of 28 days. The antibacterial inhibition assessment showed that the hydrogels have high activity against the tested bacterial strains. Due to the high cell viability, the hydrogels exhibited strong cytocompatibility. The results of the *in vitro* testing indicated the formation of fiber collagen, enhanced healing, and reduced inflammation. The hydrogels have good antibacterial properties, are biocompatible, provide improved and faster healing, and reduce inflammation. The authors explain that the main factor of the exhibited antibacterial properties is the generation of ROS when the nanoparticles interact with bacteria, which damages the bacterial cell walls, leading to reduced infections (Thanusha et al., 2018).

### 3.9 Toxicity concerns of MNPs in wound healing

While metal NPs have been widely used in treating chronic wounds and have potential antibacterial activity, some of them still exhibit toxic behaviors. The release of Ag^+^ ions by dissolution of AgNPs and oxidative stress are two primary mechanisms related to AgNPs-associated cytotoxicity (Zhang et al., 2014). Larger AgNPs have lower cytotoxicity compared to AgNPs of smaller size because of their higher specific surface area, and due to the higher release of silver ions, the cytotoxicity of AgNPs larger than 10 nm is influenced by the direct interaction with macrophage. According to *in vivo* investigations, AgNPs having a size of 10 to 100 nm and a concentration of 5 to 10 µgmL^−1^ are toxic to mitochondrial functions (Arora et al., 2009). AgNPs can influence the respiratory chain of mitochondria, generating ROS and affecting ATP and DNA damage. AgNPs are also harmful to humans. Crystallinity, shape, size, and surface chemistry are all factors that affect the cytotoxicity of AgNPs. These NPs accumulate in the testes by penetrating the blood barrier and affecting sperm cells (Reddy Panyala et al., 2008). A recent study found that AgNPs had an unfavorable influence on many stages of wound healing *in vivo*. AgNP treatment at 2 μg/ml reduced fin regeneration during epithelialization and blastema development. Cell proliferation of regenerating blastema was dramatically reduced after AgNP treatment (Pang et al., 2020).

Some types of MNPs have the ability to penetrate cells and may activate the intracellular signaling network or disrupt cell membrane, causing oxidative stress, apoptosis, and cell damage (Park et al., 2007). Some report indicated that *rainbow* trout show altered lipid peroxidation upon exposure to Cu NPs (Shaw et al., 2012). CuO NPs can diminish lysosomal function and break down lysosomal membranes in acidic conditions. Kumbhakar et.al reported larger levels of cytotoxicity on the impact of CuO NPs on meiotic and mitotic phases (Sajjad et al., 2023). Certain sizes of (40 nm, 80 nm) specific lower concentrations (1-1 mM) increased the endothelial and fibroblast proliferation. Smaller NPs (20 nm) and higher concentrations reduced the cell proliferation (Alizadeh et al., 2019).

Studies show that the toxicity of Au NPs is more affected by their surface coating compared to having biodistribution. It has been reported that there were a size-dependent and surface charge distribution of NPs in rats after being exposed to AuNPs at 5.3 μg/rat (Hir et al., 2011). It has been reported that Au was found in the placenta but not in fetal organs after being exposed to 20 and 50 nm AuNPs, without indicators of damage to the placenta or fetus (Rattanapinyopituk et al., 2014). Upon intraperitoneal injection of AuNPs to rats, major changes were showed in some liver enzymes (Abdelhalim and Abdelmottaleb Moussa, 2013). AuNPs serving as biolabels, drug carriers, and biosensors induced cytotoxicity, toxicity, and hepatotoxicity to the lungs and spleen (Abdelhalim and Abdelmottaleb Moussa, 2013; Sani et al., 2021; Daraee et al., 2016).

Overdose of TiO_2_ NPs has caused a change in the functionality and disturbance in the gastric area by lowering the digestion process. Chen et al. investigated the toxicity of TiO_2_ NPs on adult mice. Increasing the time and dose of NPs results in the loss of appetite, lethargy, and tremors (Chen et al., 2009). The effect of TiO_2_ NPs on the cardiovascular function depends on the concentration of exposure and the contact time. Morphological changes formed due to TiO_2_ NPs exposure in the brain of rats and their primary cortical neuron cultures (Valentini et al., 2018).

ZnO nanoparticles are non-toxic to HDF cells and support cells proliferation. Larger ZnO nanoparticles are biocompatible non-toxic and suitable for wound healing (Kaushik et al., 2019). But still a study on ZnO NPs talks about the generation of ZnO NPs-protein corona and the related unraveling with the formation of a mimic model (Bhunia et al., 2013). Studies says that the TiO_2_ NPs cause toxicity mainly through oxidative stress. They generate ROS which may lead to DNA damage and inflammatory responses. They can affect several organs, including the liver, lungs, heart, brain, and kidneys. Smaller particles can aggregate (<200 nm) cells more easily and accumulate in tissues, and larger particles tend to remain attached to cell surfaces (Shabbir et al., 2021).

In the case of CeO_2_ NPs, no key takeaways can be drawn on their toxicity because various CeO_2_ specimens can act differently, which is probably accountable for the inconsistency reported in the literature (Leung et al., 2015).

The researchers discovered that injecting MgO NPs into Wistar rats increases the levels of liver function indicators such as ALP and AST. In rats, injection with MgO NPs resulted in no alteration in serum GGT and ALT levels, alongside renal function. In the hematology experiment, white blood cells increased notably at MgO NP doses of 250 and 500 μg.mL^-1^, indicating that these NPs cause inflammation in liver tissues (Mazaheri et al., 2019).

## 4 Discussions

MNPs have emerged as good agents in wound healing due to their antimicrobial properties and their ability to modulate oxidative stress. Incorporating these nanomaterials into smart hydrogels will enhance the efficacy and toxicity. As a highly moisturizing material, hydrogels have outstanding adhesion and degradation properties. The performance of hydrogels against the *Staphylococcus aureus*, *Escherichia coli* bacteria strain was assessed, and potentially proved their antibacterial activity (Ahsan and Farooq, 2019; Mahmoud et al., 2019; Khorasani et al., 2018). Integrating NPs into hydrogels improved their structural and biological properties by accelerating the wound healing process, reducing inflammation, enhancing mechanical properties, promoting cell proliferation, tissue regeneration, and attachment (Akturk et al., 2016; Abbas et al., 2024; Rahmanpour et al., 2022; Blinov et al., 2023). Apart from having a handful of merits, the authors explain that the long-term safety of such nanocomposites in clinical settings needs further testing and exploration to ensure their biocompatibility and effectiveness when used for various types of wounds. The authors specifically do not address the scalability of such dressings and their production cost for wide clinical use (Akturk et al., 2016).

A new study in metal-embedded wound dressings allows the therapeutics to go beyond the antimicrobial effects towards metabolic reprogramming. A new therapeutic strategy was developed against the Diabetic foot ulcer pathogens (DFU) utilizing metal-polyphenol NPs fabricated from ferric ions (Fe^3+^) and Epigallocatechin-3-gallate (EGCG). These NPs were later integrated with glucose oxidase (GO_x_) and salvianolic acid B (SAB) to improve their multiple biological effects. This innovative system showed enhanced antibacterial effectiveness against pathogens that are associated with Diabetic foot ulcer (Yang et al., 2025). He et al. developed an artificial cascade-nanozyme-loaded hydrogel dressing to accelerate the healing of DFUs. This hydrogel dressing is capable of ROS scavenging and oxygenation, and therefore significantly reduce the M1/M2 ratio of macrophages, enhance oxygenation efficiency, and decrease the inflammatory level of DFUs, hence, generating a suitable microenvironment for macrophage control and angiogenesis (He et al., 2025). Smart hydrogels are used to treat chronic infectious diabetic wounds. In this research, a hydrogel dressing with anti-inflammatory and bactericidal properties was created by fabricating a pH- and ROS-responsive scaffold utilizing 4-arm PEG-dopamine and hyaluronic acid modified with phenylboronic acid, depending on dynamic borate ester linkages. This was subsequently incorporated with an antimicrobial peptide (AMP), and a ROS-sensitive micelle, mPEG-TK-PLGA, filled with quercetin (QC). In this study, they conducted a comprehensive evaluation of the hydrogel’s mechanical and physical properties, its antibacterial and anti-inflammatory capabilities, and explored the underlying mechanisms of action, demonstrating the hydrogel’s significant therapeutic potential for treating diabetic wounds (Li et al., 2024).

## 5 Limitations

The results in this review indicate that MNPs in wound patches offer various significant benefits, such as accelerating wound closure, inhibiting bacteria from growing, and decreasing inflammation responses. However, there are certain aspects that limit the utilization of such nanomaterials that need to be considered. For instance, MNPs such as Au NPs and Ag NPs show some cytotoxicity against human skin fibroblasts. The cytotoxicity is found to be higher when the MNPs used are in spherical shape, compared to when utilizing rod-shaped MNPs (Favi et al., 2015). Such a limitation needs to be addressed crucially as the cytotoxicity of some types of MNPs can be due to other issues, including cell damage, oxidative stress accumulation, and inhibition of cell proliferation (Xiong et al., 2022).

Also, the utilization of MNPs in wound healing relies heavily on their own mechanism in inhibiting bacteria, while using them as carriers for drug delivery to wounds has several limitations, including the non-uniform release of the encapsulated drug into the wound region and insufficient drug delivery into the targeted region. These limitations mainly arise due to the chemical and physical properties of MNPs (Xu et al., 2023). These limitations need to be addressed to ensure sufficient effect on the wound healing process. Such limitations can be addressed by combining the MNPs system with macrophage coating, where drug-loaded MNPs are coated with macrophages, and this leads to enhanced wound healing effects (Xu et al., 2023). Also, further exploring the mechanisms that nanoparticles use to enhance wound healing is necessary, as it enables the development of more effective and targeted nanoparticle-based wound treatment. Furthermore, there is a main focus on using Au NPs and Ag NPs in bacteria elimination and wound healing, while other types of MNPs have relatively limited background and testing in this field. Hence, it is necessary to expand the exploration of the performance of different types of MNPs that could be cheaper and broadly available.

While most studies focus on testing the patches on mice and rats, further studies need to include testing on actual human skin. This is because mice and rats have physiological and structural differences compared to human skin, in addition to differences in the skin wound healing mechanism (Masson‐Meyers et al., 2020). Also, the majority of *in vivo* tests conducted by studies focus on the *E. coli*, *S. aureus*, and *Pseudomonas aeruginosa* strains, while other strains need to be tested. For instance, chronic wound infections involve other strains such as *Enterococcus faecalis, coagulase-negative staphylococci, Proteus* spp.*, and anaerobic bacteria* (Serra et al., 2015). Moreover, it would be beneficial to have a control sample of liquid exposure for biocompatible MNPs, where a solution of MNPs is directly injected into the wound to observe the full effect of MNPs in wound healing.

## 6 Conclusion

This review was conducted to explain and assess the performance of different types of MNPs in bacteria reduction, inflammation reduction, and overall wound healing when incorporated into wound healing patches. The underlying mechanisms responsible for the elimination of bacteria and how MNPs interact with bacteria at the wound site were discussed and explained thoroughly. The existing literature on this topic indicates that various MNPs exhibit antibacterial and anti-inflammatory properties, and the most common mechanisms of bacterial elimination are the ROS generation and the release of metallic ions. The elimination of bacteria occurs due to several factors caused by the MNPs, such as disrupting their cell walls and starvation of nutrients and growth factors. Also, it was found that the presence of MNPs on the wound site offers several benefits, including the acceleration of the healing process, wound closure, and cell proliferation.

While MNPs offer several benefits in wound healing, there is a gap in the assessment of the long-term toxicity effects of nanoparticles and exposure to metal ions. Also, long-term exposure to such nanomaterials could cause immune responses that need to be taken into consideration. Moreover, MNPs other than Au NPs and Ag NPs need to be further explored and tested, as information about their bacterial elimination mechanisms, effectiveness, and background is limited.
